# Real-World Effectiveness and Safety of Upadacitinib and Abrocitinib in Moderate-to-Severe Atopic Dermatitis: A 52-Week Retrospective Study

**DOI:** 10.3390/jcm14092953

**Published:** 2025-04-24

**Authors:** Luciano Ibba, Costanza Falcidia, Sara Di Giulio, Matteo Bianco, Mario Valenti, Paola Facheris, Alessandra Narcisi, Antonio Costanzo, Luigi Gargiulo

**Affiliations:** 1 Dermatology Unit, IRCCS Humanitas Research Hospital, Rozzano, 20089 Milan, Italy; costanza.falcidia@humanitas.it (C.F.); sara.digiulio@humanitas.it (S.D.G.); matteo.bianco@humanitas.it (M.B.); mario.valenti@hunimed.eu (M.V.); paola.facheris@humanitas.it (P.F.); alessandra.narcisi@hunimed.eu (A.N.); antonio.costanzo@hunimed.eu (A.C.); luigi.gargiulo@humanitas.it (L.G.); 2 Department of Biomedical Sciences, Humanitas University, Pieve Emanuele, 20072 Milan, Italy

**Keywords:** atopic dermatitis, upadacitinib, abrocitinib

## Abstract

**Background:** Atopic dermatitis (AD) is a chronic pruritic inflammatory disease affecting children and adults. Upadacitinib and abrocitinib are selective Janus kinase 1 inhibitors approved for the treatment of moderate-to-severe AD. Although their efficacy and safety are described in phase 3 clinical trials, real-world data are limited. **Objectives:** We aimed to evaluate the effectiveness and safety of upadacitinib and abrocitinib treatment in a real-life adult population with moderate-to-severe AD throughout an extended observation period. **Methods:** This retrospective observational study was conducted by analyzing data from the electronic records of IRCCS Humanitas Research Hospital from January 2023 to December 2024. Patients were administered either upadacitinib (15 or 30 mg) or abrocitinib (100 or 200 mg). Effectiveness was evaluated by using clinician-reported scores (Investigator Global Assessment [IGA] and Eczema Area and Severity Index [EASI]) and patient-reported outcomes (peak pruritus numerical rating scale [PP-NRS]) at weeks 8, 16, 32 and 52. Statistical significance was set at a probability value (*p*-value) < 0.05. Adverse events were also collected. **Results:** In total, 129 patients were included in the study, and 84 of them reached 52 weeks. At week 52, the EASI 75, 90, and 100 responses were 88.9%, 70.8%, and 54.2% for upadacitinib, and 100%, 91.7%, and 75% for abrocitinib. An IGA score equal to 0 or 1 at 52 weeks was achieved by 84.7% of patients treated with upadacitinib and 100% of those receiving abrocitinib. A four-point reduction from baseline PP-NRS was reported by 86.1% for upadacitinib and by 83.3% of patients for abrocitinib after one year of follow-up. **Conclusions:** Our study showed comparable or even higher effectiveness outcomes in terms of EASI 75, EASI 90, and EASI 100 at week 52 compared to phase-3 clinical trials, with no new safety concerns, supporting the real-world effectiveness of abrocitinib and upadacitinib in moderate-to-severe AD.

## 1. Introduction

Atopic dermatitis (AD) is a chronic, pruritic inflammatory skin disease that affects infants, children, adolescents, and adults worldwide. AD is often associated with elevated serum immunoglobulin (IgE) levels and a personal or family history of eczema, allergic rhinitis, and/ or allergic asthma [1]. Approximately 15–20% of children and 1–3% of adults worldwide are affected [2,3].

The pathophysiology of AD is multifactorial, involving genetic predisposition, dysfunctional skin barriers, microbiome abnormalities, immune dysregulation, and environmental triggers [4]. The FLG gene encoding filaggrin is a major genetic factor in AD. Loss-of-function mutations in FLG are strongly associated with AD, especially in Europe and Asia, and increase susceptibility to asthma and peanut allergies [4,5]. Environmental factors such as pollution, tobacco smoke, and hygiene practices contribute to AD, with higher risk in particular in metropolitan areas [6].

Immunologically, AD is characterized by type-2 immune dysregulation, with proinflammatory cytokines like IL-4 and IL-13 playing a central role. In particular, both of these cytokines contribute to the onset and development of AD pathogenesis by signaling through Janus kinases (JAKs) and activating the signal transducer and activator of transcription-6 (STAT6), a key transcription factor in their biological activity [7]. At the same time, skin barrier defects and microbial dysbiosis (e.g., Staphylococcus aureus overgrowth) can exacerbate the condition [4].

The “itch–scratch cycle” is a hallmark of the disease, with scratching damaging the skin, allowing other allergens to enter and triggering inflammation and, therefore, pruritus [8].

Clinically, AD presents with dryness, severe pruritus, erythematous lesions, and thickened skin due to chronic scratching. The disease varies by age, with infants typically showing lesions on the face and diaper area, while children may develop flexural lesions. In adults, AD can be more diffuse and involve lichenification and nodular prurigo [4,8]. Comorbidities associated with AD include asthma, hay fever, food allergies, and mental health issues such as anxiety and depression [9,10].

The management of AD is based on the severity of the disease and requires a multimodal approach involving patient and family education. This has been shown to greatly improve quality of life when combined with skin hydration, pharmacologic treatment, and elimination of exacerbating factors [11,12].

For moderate-to-severe AD, systemic drugs are preferred. The European Guidelines of Atopic Eczema recommended six different drugs including dupilumab, tralokinumab and three JAK inhibitors (upadacitinib, abrocitinib and baricitinib) [11]. In particular, JAK inhibitors have shown efficacy and safety in both clinical trials and real-world experiences, significantly improving cutaneous lesions and patients’ symptoms with faster responses compared to monoclonal antibodies [13,14,15,16,17]. In particular, abrocitinib is an oral JAK1 selective inhibitor approved for the treatment of moderate-to-severe AD at 100 mg and 200 mg daily doses [18]. Abrocitinib has demonstrated efficacy in several phase-III clinical trials compared with both placebo and dupilumab [19,20]. Common side effects reported were self-limiting nausea in the first days of therapy, elevated serum creatine phosphokinase (CPK), and a dose-related decrease in platelet count [19,20]. Upadacitinib also selectively and reversibly inhibits JAK1, and it was approved for the treatment of moderate-to-severe AD across two dosages (15 mg and 30 mg), after being evaluated in several phase-III clinical trials, demonstrating superiority over both the placebo and dupilumab [21,22,23]. The most commonly reported adverse effects were acne, upper respiratory tract infections (URTIs), nasopharyngitis, headache, and elevation of CPK [22,23]. Despite the large use of JAK inhibitors in clinical practice, long-term experiences in this setting are limited. To address this unmet need, we conducted a 52-week retrospective monocentric study to evaluate the effectiveness and safety profile of the two selective JAK1 inhibitors, upadacitinib at a dosage of 15 or 30 mg and abrocitinib at a dosage of 100 or 200 mg, in patients with severe AD who received continuous treatment for at least one year.

## 2. Materials and Methods

This retrospective study was carried out by analyzing the AD database records of IRCCS Humanitas Research Hospital-Rozzano (Milan) between January 2023 and December 2024.

We enrolled a total of 129 patients. Eligibility for treatment with upadacitinib and abrocitinib was determined in accordance with the latest European Guidelines for the systemic management of atopic dermatitis in adults [11]. All patients received either upadacitinib at doses of 15 mg or 30 mg daily or abrocitinib at doses of 100 mg or 200 mg, as per their Summary of Product Characteristics [18,21]. According to the Agenzia Italiana del Farmaco (AIFA), choosing between upadacitinib 15 mg and 30 mg or between abrocitinib 100 mg and 200 mg is based on the physician’s discretion. In our clinical practice, patients were prescribed either the higher or lower dosage based on disease severity, while patients ≥ 65 years old all received upadacitinib 15 mg or abrocitinib 100 mg. Before the start of treatment with either upadacitinib or abrocitinib, a wash-out period of at least 4 weeks was advised for patients who had been using previous systemic therapies for the management of AD. Patient demographics, comorbidities, disease profile and previous therapeutic strategies were extracted from the electronic medical records. During each visit, assessments were conducted using established scales such as the EASI (Eczema Area and Severity Index) score, the IGA (Investigator Global Assessment) score, the peak pruritus (PP)-NRS (Numerical Rating Scale) score and the sleep-NRS score at baseline, week 8, week 16, week 32 and week 52. The effectiveness of upadacitinib and abrocitinib was evaluated at each predefined time point by determining the percentages of patients achieving 75%, 90%, and 100% (EASI 75, EASI 90, and EASI 100) improvements in EASI compared to the baseline EASI. Moreover, we determined the percentages of patients reaching an IGA score of 0/1 (indicating clear or almost clear skin) and an absolute EASI score equal to or less than 7. Regarding patient-reported outcomes, we assessed the reduction of at least 4 points in the PP-NRS compared to the baseline and an absolute PP-NRS of 0/1. These effectiveness endpoints were selected based on those utilized in pivotal phase-3 clinical trials for both drugs [22]. Documentation regarding prior use, or lack thereof, of systemic corticosteroids or dupilumab before starting the JAK inhibitor was recorded, along with the reason for discontinuation. Safety was monitored according to reported adverse events (AEs), including serious AEs, AEs leading to discontinuation, AEs requiring dosage modification, and laboratory value abnormalities (hematology, clinical chemistry, and urinalysis). Serious AEs were defined as events leading to death, hospitalization (or the prolongation of hospitalization), life-threatening AEs, AEs resulting in persistent or significant disability/ incapacity, or AEs causing congenital anomalies/ birth defects [24]. The occurrence of AEs was collected at each time point. Due to the study’s retrospective nature, not all the patients were seen for a full 52 weeks. Data for any unattended follow-up visits were considered missing.

The statistical analysis was rigorously conducted, aligning with the intention-to-treat principle, encompassing the entire cohort of 129 patients administered either upadacitinib or abrocitinib. Statistical analysis was performed using Stata/SE 18.0. Tables and Figures were generated using Microsoft Excel and GraphPad Prism 10.2. Continuous variables were summarized using mean and standard deviation (SD), while categorical variables were presented as absolute numbers and percentages.

To assess differences in terms of effectiveness and safety outcomes between upadacitinib and abrocitinib, categorical variables were compared using the chi-square test, while continuous variables were analyzed using Student’s *t*-test or the Mann–Whitney U test when parametric assumptions were not met. Statistical significance was determined by a probability value (*p*-value) less than 0.05, denoting substantial findings.

Institutional review board approval was exempted as the study protocol did not deviate from standard clinical practice. All patients received abrocitinib or upadacitinib as in good clinical practice, in accordance with European guidelines. All included patients had provided written consent for a retrospective study of data collected during routine clinical practice (demographics, clinical scores). This study was performed in accordance with the Helsinki Declaration of 1964 and its later amendments. Data collection and handling complied with applicable laws, regulations, and guidance regarding patient protection, including patient privacy.

## 3. Results

### 3.1. Study Population

This monocentric retrospective study enrolled 129 patients, of whom 104 received upadacitinib (15 mg or 30 mg) and 25 received abrocitinib (100 mg or 200 mg). All patients completed at least 16 weeks of treatment. Additionally, 86 and 74 patients in the upadacitinib group and 18 and 12 in the abrocitinib group completed 32 weeks and 1 year of follow-up, respectively. Demographic characteristics were comparable between the two groups regarding both patients’ features and disease severity at baseline (Table 1).

In the upadacitinib cohort, 52 patients were males (50%), and 90 (86.5%) exhibited involvement of at least one sensitive area, including the face/neck, hands, and genitalia, at baseline. Similarly, in the abrocitinib cohort, 14 patients were males (56%), and 18 (72%) had the involvement of at least one sensitive area.

The mean age was comparable between patients receiving upadacitinib (36.65 years [SD 16.30]) and those receiving abrocitinib (39.56 years [SD 15.86]). Similarly, no significant differences were observed between the cohorts in terms of mean BMI, PP-NRS, and baseline EASI score. Specifically, the mean BMI (Body Mass Index) in the upadacitinib group was 23.84 (SD 3.46), the PP-NRS was 7.58 (1.85 SD), and the mean baseline EASI score was 19.22 (SD 7.22). Correspondingly, patients treated with abrocitinib had a mean BMI of 23.44 (SD 2.29), a PP-NRS value of 7.24 (SD 1.96), and a mean baseline EASI score of 19.15 (SD 6.57).

Disease duration was slightly longer in patients treated with upadacitinib compared to those treated with abrocitinib (23.45 years [SD 16.67] vs. 17.80 years [SD 13.32]).

Concerning the severity of AD, patients started with a mean baseline EASI of 19.22 (SD 7.22) and 19.15 (SD 6.57) for patients taking upadacitinib and abrocitinib, respectively. Although not statistically significant, patients receiving abrocitinib were more likely to have severe AD (defined as an IGA score of 4) compared to those on upadacitinib [11 patients (44%) vs. 35 patients (33.7%)]. The PP-NRS score at baseline was 7.58 (SD 1.85) and 7.24 (SD 1.96) in the cohort of patients treated with upadacitinib and abrocitinib, respectively.

Atopic comorbidities, including allergic rhinitis, allergic conjunctivitis, asthma, or food allergy, were slightly more frequent in patients receiving upadacitinib compared to those receiving abrocitinib (37.5% vs. 24%).

A history of previous biological treatment with dupilumab was reported in 41.4% of patients in the upadacitinib group, and 36% of those in the abrocitinib group reported a history of previous biological treatment with dupilumab. Among the total of 52 patients who switched their therapy from dupilumab to a JAK inhibitor, the most common reason was secondary ineffectiveness (30 patients), followed by primary ineffectiveness (14 patients) and AEs in 8 patients (7 recurrent conjunctivitis and 1 arthralgia).

Among patients receiving upadacitinib, most (92.3%) exhibited the classic AD phenotype, while 3.8% of the patients showed prurigo nodularis-like AD. In contrast, all patients treated with abrocitinib presented with the classical AD phenotype.

The additional characteristics at baseline of both groups are shown in Table 1.

### 3.2. Effectiveness

**Figure 1:**
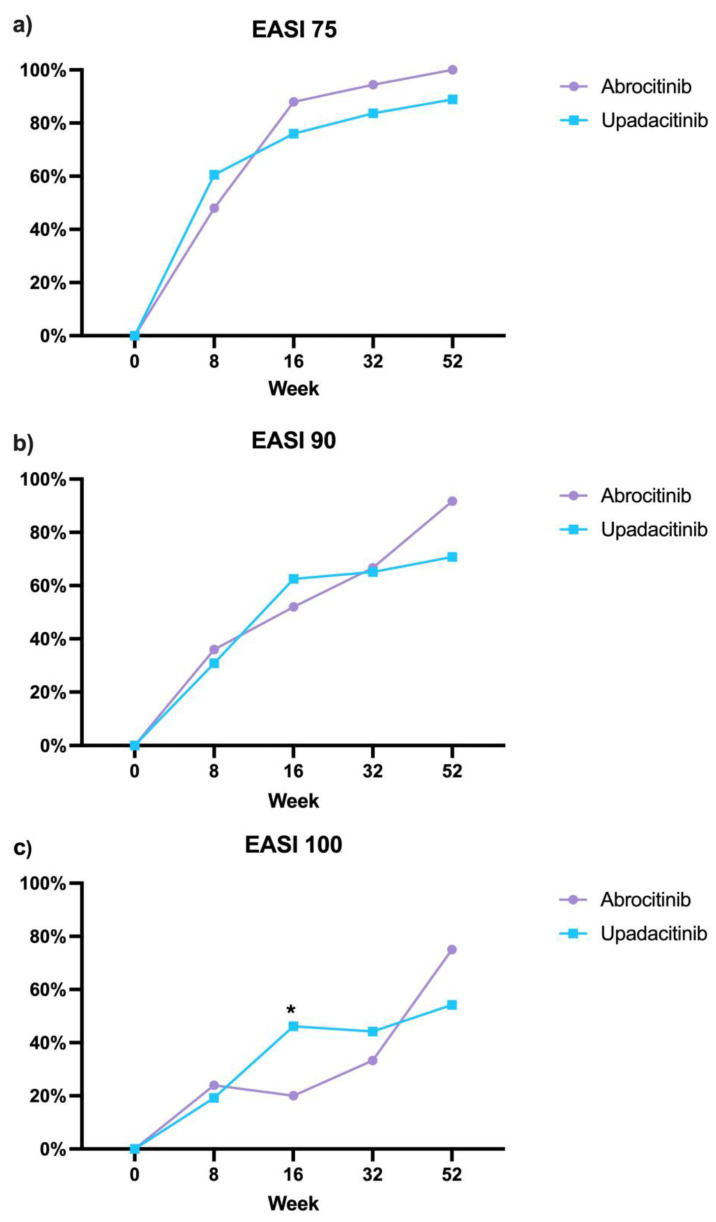
Effectiveness in terms of EASI 75 (**a**), 90 (**b**), 100 (**c**) in patients treated with abrocitinib and upadacitinib at weeks 8, 16, 32 and 52. EASI: Eczema Area and Severity Index; * *p* < 0.05.

Among the 104 patients treated with upadacitinib, after 8 weeks of treatment, EASI 75, EASI 90, and EASI 100 were achieved by 60.6%, 30.8%, and 19.2% of patients, respectively. At the same time point, these response rates were 48%, 36%, and 24% in patients receiving abrocitinib (Figure 1). After 16 weeks of treatment, 76% and 62.5% of patients achieved EASI 75 and EASI 90. Similarly, 88% and 52% of patients treated with abrocitinib achieved EASI 75 and EASI 90, respectively. Patients treated with abrocitinib at the same time point were less likely to achieve complete skin clearance (EASI 100) compared to those treated with upadacitinib (20% vs. 46.2%, *p* = 0.017) (Figure 1). After one year of follow-up, 88.9%, 70.8%, and 54.2% of patients in the upadacitinib group reached EASI 75, EASI 90, and EASI 100, respectively. In comparison, among the 12 patients receiving abrocitinib, they all reached EASI 75, while 91.7% achieved EASI 90, and 75% reached complete skin clearance (Figure 1, Table 1).

**Figure 2:**
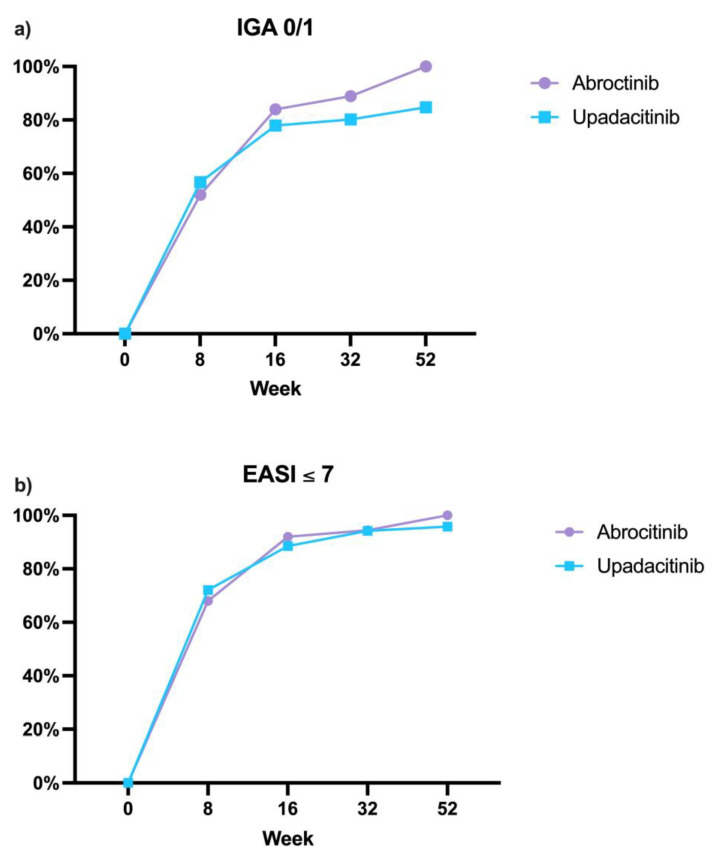
Percentage of patients treated with abrocitinib and upadacitinib achieving absolute outcomes of EASI ≤ 7 (**a**) and IGA 0/1 (**b**) at weeks 8, 16, 32, and 52 of follow-up. EASI: Eczema Area and Severity Index; IGA: Investigator Global Assessment.

Regarding the other effectiveness outcomes, there were no statistically significant differences between the two cohorts in terms of absolute EASI score ≤ 7 and IGA score 0/1 throughout the study period (Figure 2). EASI ≤ 7 was recorded in 75 (72.1%), 92 (88.5%), 81 (94.2%), and 69 (95.8%) patients at weeks 8, 16, 32, and 52, respectively, in the upadacitinib group. Similarly, in the abrocitinib group, 17 (68%), 23 (92%), 17 (94.4%), and 12 (100%) patients reached this outcome at the same time points. An IGA of 0 or 1 (clear or almost clear) was achieved by 56.7% at week 8, 77.9% at week 16, 80.2% at week 32 and 84.7% after one year of treatment with upadacitinib. At each of the same weeks, these endpoints were reached by 52%, 84%, 88.9%, and 100% of those treated with abrocitinib (Figure 2, Table 2).

**Figure 3:**
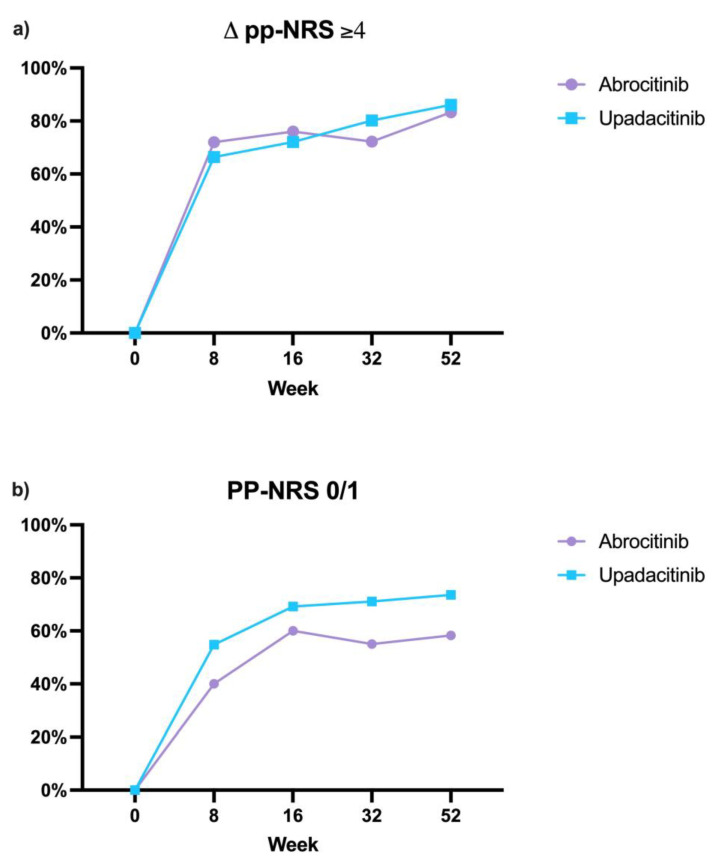
Clinical improvement in the two cohorts of patients based on Patient-Reported Outcomes: PP-NRS reduction ≥ 4 points (**a**) and PP-NRS of 0 or 1 (**b**). PP-NRS: peak pruritus numerical rating scale.

Regarding the patient-reported outcomes (PROs), we assessed the effectiveness of upadacitinib and abrocitinib based on the achievement of a PP-NRS score of 0/1 and on the reduction in the same score of at least four points compared with the baseline. In particular, in the upadacitinib cohort, the percentage of patients reaching a PP-NRS score of 0/1 was 54.8% at week 8, rising to 69.2% at week 16, 71.1% at week 32, and 73.6% at week 52. In addition, a reduction of at least four points was recorded in 66.4% of patients at week 8, increasing to 72.1% at week 16, 80.2% at week 32, and 86.1% at week 52. Similarly, in the abrocitinib group, 40% of patients achieved a PP-NRS score of 0 or 1 at week 8, reaching 60% at week 16, 55% at week 32, and 58.3% at week 52. The proportion of patients experiencing a reduction of at least four points was 72% at week 8, 76% at week 16, 72.2% at week 32, and 83.3% at week 52 (Figure 3, Table 2).

### 3.3. Safety

Our study showed comparable safety profiles between the two groups of patients. No serious AEs were observed throughout the study period for both groups (Table 3).

Regarding patients treated with upadacitinib, the most frequently reported AEs included weight gain (18 patients), followed by microcomedonal acne (7 patients), hypercholesterolemia (5 patients), and herpes zoster infection (4 patients). In our study, a total of two patients discontinued upadacitinib due to AEs (one patient for hypercholesterolemia and one patient for reactivation of herpes zoster). In addition, one patient interrupted the drug because of an accidental pregnancy (Table 3).

In the abrocitinib group, acne and hypercholesterolemia were the most commonly reported AEs, involving two patients each. Only one patient discontinued abrocitinib due to impetigo (Table 3). In both treatment groups, no new safety findings were observed.

## 4. Discussion

This study confirmed the effectiveness and safety of upadacitinib and abrocitinib throughout 52 weeks of observation in a real-world adult population affected by moderate-to-severe AD, which represents one of the largest and longest reported real-world populations to date [25,26,27,28,29,30,31,32]. Our study shows similar or even higher rates of EASI 75, EASI 90, and EASI 100, compared to data from clinical trials [19,20,22,23]. In particular, in the phase-3 Measure Up-1 and Measure Up-2 trials at week 52, an EASI 75 was achieved by 82% and 79.1% of patients receiving upadacitinib 15 mg and 84.9% and 84.3% of patients treated with upadacitinib 30 mg as monotherapy (for Measure Up-1 and Measure Up-2, respectively) [22]. In the AD Up study, patients were allowed to concomitantly apply topical corticosteroids, as in clinical practice. This clinical trial determined the rate of EASI 75 after one year to be 77.1% and 64.6% for upadacitinib 30 mg and 15 mg, respectively [23]. In our study, EASI 75 at week 52 was reached by a higher percentage of patients (88.9%) taking upadacitinib in a dose of either 15 mg or 30 mg. Regarding other real-world experiences, our results are consistent with most of the studies including 52 weeks of follow-up [31]. In a recent real-world study, *Gargiulo* et al. evaluated the effectiveness of upadacitinib 30 mg in a population of 31 patients. After one year of treatment, 84% and almost 70% of patients achieved EASI 90 and EASI 100, respectively [33]. Regarding abrocitinib, real-world data are currently limited [25,26]. The JADE MONO-1 clinical trial followed patients taking abrocitinib as monotherapy for 12 weeks and showed an EASI 75 of 40% for abrocitinib 100 mg and 63% for abrocitinib 200 mg [19]. Similarly, in our study, EASI 75 was achieved by 48% and 88% of patients after 8 and 16 weeks of follow-up, respectively. At week 52, the same outcome was reached by 100% of patients taking either abrocitinib 100 mg or 200 mg. Compared to a recent 24-week Spanish real-world study, we observed higher rates of EASI 75 at week 32 (70.6% vs. 94.4%) [25]. In our study, after 16 weeks of treatment with abrocitinib, EASI 90 was achieved by 52% of patients. This result is consistent with another real-world multicenter Italian study in which almost half of the patients (49.4%) achieved EASI 90 after 16 weeks of treatment with abrocitinib [26]. In our experience, we observed similar clinical responses in the two groups, the only significant difference being the percentage of patients achieving EASI 100 at week 16 (Table 2, Figure 2). This result is consistent with recent network meta-analyses that found higher responses at week 16 for patients receiving upadacitinib [34]. In our study, we did not perform a sub-analysis to identify predictors of better response to JAK inhibitors due to the limited sample size. However, recent real-world studies have investigated the role of clinical variables associated with higher effectiveness in AD patients treated with JAK inhibitors [35,36,37]. In particular, Hagino et al. found that a lower baseline head/neck EASI score, a lower systemic inflammatory response index (SIRI), and the absence of bronchial asthma may be associated with better outcomes in patients treated with upadacitinib [35]. In addition, patients with lower baseline IgE levels may respond more favorably to upadacitinib, suggesting a possible inverse relationship between IgE concentration and treatment response [36]. Elevated total serum IgE is a hallmark of atopy and reflects the degree of Th2-mediated immune activation [36]. Although formal data are currently lacking, it has been hypothesized that patients with less severe disease at baseline (e.g., IGA ≤ 3 or lower EASI) may have a more favorable response to JAK inhibitors than those with more severe disease, possibly due to a lower inflammatory threshold.

The safety profile of both drugs was consistent with those reported in other real-world studies and the data from clinical trials [38,39,40,41,42]. Both JAK inhibitors also showed dose flexibility, as in 18 patients (14%), the dose was escalated or reduced to meet the patient’s clinical needs. For both drugs, the most common clinical AEs were acne and weight gain. A recent study highlighted the correlation between weight gain and JAK inhibitors, focusing in particular on the role of leptin mediated by JAK2. The increased dosage of upadacitinib to 30 mg and its subsequent action on JAK2 too could explain the high frequency of this AE in our patient cohort as well [43,44,45]. Recent real-world studies have demonstrated the safety of this class of drugs, even in high-risk populations [42,46].

Our study has several limitations, including its retrospective design, the limited cohort of patients treated with abrocitinib, especially at week 52, and the probable underestimation of mild-to-moderate AEs due to its real-world nature. Furthermore, because of the limited number of patients in each dose subgroup, dose-stratified analyses could not be conducted. Given the retrospective nature of the study, selection bias may be present, and propensity score matching was not performed. Although baseline characteristics were comparable between treatment groups, which helped mitigate risk, the possibility of unmeasured confounding variables cannot be ruled out. However, this is one of the largest studies to date on JAK inhibitors in AD patients with a 52-week follow-up, and it provides more evidence of the sustained long-term response achieved by both upadacitinib and abrocitinib. Despite a slightly higher EASI 100 rate at week 16 in patients treated with upadacitinib, both drugs achieved significant clinical responses throughout the year of observation. Patients who did not respond to conventional systemic agents or biologics also achieved this satisfactory response, highlighting the effectiveness of abrocitinib and upadacitinib. Larger prospective studies are needed in clinical practice to further establish the role of these drugs in the long-term management of AD.

## Figures and Tables

**Table 1:** Demographic and disease characteristics of the two cohorts of patients at baseline.

	Abrocitinib (n = 25)	Upadacitinib (n = 104)	
	**N (%)**		***p*-Value**
**Male**	14 (56)	52 (50)	0.29
***AD Phenothype***			
**Classic**	25 (100)	96 (92.3)	NA
**Prurigo nodularis-like**		4 (3.8)	NA
**Nummular eczema**		2 (1.9)	NA
**Generalized inflammatory**		2 (1.9)	NA
**At least one AD sensitive area**	18 (72)	90 (86.5)	0.077
**Bio-Experienced**	9 (36)	43 (41.4)	0.625
**At least one cardiometabolic comorbidity**	6 (24)	20 (19.2)	0.594
**At least one atopic comorbidity**	6 (24)	39 (37.5)	0.203
**IGA = 4 at baseline**	11 (44)	35 (33.7)	0.322
	**Mean (SD)**		***p*-Value**
**Age, years**	39.56 (15.86)	36.65 (16.30)	0.979
**BMI, kg/m^2^**	23.44 (2.29)	23.84 (3.46)	0.646
**Disease duration, years**	17.80 (13.32)	23.50 (16.67)	0.08
**mean EASI at baseline**	19.15 (6.57)	19.22 (7.22)	0.966
**PP-NRS at baseline**	7.24 (1.96)	7.58 (1.85)	0.421

AD: atopic dermatitis; IGA: Investigator Global Assessment; SD: standard deviation; BMI: Body Mass Index; EASI: Eczema Area and Severity Index; PP-NRS: peak pruritus numerical rating scale; NA: Not Applicable.

**Table 2:** Detailed data on treatment effectiveness, including relative, absolute, and patient-reported outcomes in patients treated with abrocitinib and upadacitinib.

	Abrocitinib	Upadacitinib	
**Week 8**	**N (%)**		***p*-Value**
EASI 75	12/25 (48)	63/104 (60.6)	0.252
EASI 90	9/25 (36)	32/104 (30.8)	0.614
EASI 100	6/25 (24)	20/104 (19.2)	0.594
IGA 0/1	13/25 (52)	59/104 (56.7)	0.669
EASI ≤ 7	17/25 (68)	75/104 (72.1)	0.683
Δ pp-NRS ≥ 4	18/25 (72)	69/104 (66.4)	0.588
PP-NRS 0/1	10/25 (40)	57/104 (54.8)	0.183
**Week 16**	**N (%)**		***p*-Value**
EASI 75	22/25 (88)	79/104 (76)	0.19
EASI 90	13/25 (52)	65/104 (62.5)	0.335
EASI 100	5/25 (20)	48/104 (46.2)	0.017
IGA 0/1	21/25 (84)	81/104 (77.9)	0.5
EASI ≤ 7	23/25 (92)	92/104 (88.5)	0.61
Δ pp-NRS ≥ 4	19/25 (76)	75/104 (72.1)	0.695
PP-NRS 0/1	15/25 (60)	72/104 (69.2)	0.376
**Week 32**	**N (%)**		***p*-Value**
EASI 75	17/18 (94.4)	72/86 (83.7)	0.239
EASI 90	12/18 (66.7)	56/86 (65.1)	0.9
EASI 100	6/18 (33.3)	38/86 (44.2)	0.397
IGA 0/1	16/18 (88.9)	69/86 (80.2)	0.387
EASI ≤ 7	17/18 (94.4)	81/86 (94.2)	0.966
Δ pp-NRS ≥ 4	13/18 (72.2)	69/86 (80.2)	0.449
PP-NRS 0/1	10/18 (55)	62/86 (71.1)	0.167
**Week 52**	**N (%)**		***p*-Value**
EASI 75	12/12 (100)	64/72 (88.9)	0.225
EASI 90	11/12 (91.7)	51/72 (70.8)	0.129
EASI 100	9/12 (75)	39/72 (54.2)	0.177
IGA 0/1	12/12 (100)	61/72 (84.7)	0.146
EASI ≤ 7	12/12 (100)	69/72 (95.8)	0.471
Δ pp-NRS ≥ 4	10/12 (83.3)	62/72 (86.1)	0.799
PP-NRS 0/1	7/12 (58.3)	53/72 (73.6)	0.278

EASI: Eczema Area and Severity Index; PP-NRS: peak pruritus numerical rating scale; IGA: Investigator Global Assessment.

**Table 3:** Safety profile of abrocitinib and upadacitinib throughout the study period.

	Abrocitinib	Upadacitinib
	**N(%)**	
**Total AEs**	8 (32)	44 (42.3)
Weigh gain	1 (4)	18 (17.3)
Acne	2 (8)	7 (6.7)
Hypercholesterolemia	2 (8)	5 (4.8)
Herpes zoster	1 (4)	4 (3.9)
URTIs	0	3 (2.9)
Anemia	0	2 (1.9)
Asthenia	0	2 (1.9)
Herpes Simplex Infection	1 (4)	2 (1.9)
Gastrointestinal symptoms	0	1 (0.9)
Impetigo	1 (4)	0
**Patients with SAEs**	0	0
**Permanent discontinuations due to AEs**	1 (4)	2 (1.9)

AE: adverse event; URTI: upper respiratory tract infection; SAE: serious adverse event.

## Data Availability

Additional data supporting the findings of this manuscript are available on reasonable request to the corresponding author.

## Abbreviations

The following abbreviations are used in this manuscript:

EASI	Eczema Area and Severity Index
IGA	Investigator Global Assessment
PP-NRS	Pruritus Numerical Rating Scale
AD	Atopic dermatitis
